# Willingness to pay and price elasticity of demand for long-acting injectable cabotegravir among men who have sex with men in Guangxi, China

**DOI:** 10.3389/fphar.2024.1367830

**Published:** 2024-10-15

**Authors:** Shiwen Chen, Yuhua Ruan, Lu Liu, Hengyan Pei, Yu Jiang, Tengda Huang, Yuxia Wei, Litai Qin, Xuebin Dai, Yu Liu, Junhui Liu, Yihong Xie

**Affiliations:** ^1^ Department of Epidemiology and Biostatistics, School of Public Health, Guangxi Medical University, Nanning, China; ^2^ State Key Laboratory of Infectious Disease Prevention and Control (SKLID), National Center for AIDS/STD Control and Prevention (NCAIDS), Chinese Center for Disease Control and Prevention (China CDC), Collaborative Innovation Center for Diagnosis and Treatment of Infectious Diseases, Beijing, China; ^3^ Division of AIDS Control and Prevention, Nanning Center for Disease Control and Prevention, Nanning, China

**Keywords:** pre-exposure prophylaxis, long-acting injectable cabotegravir, men who have sex with men, willingness to pay, price elasticity of demand

## Abstract

**Objectives:**

There is still no study focused on willingness to pay for long-acting injectable cabotegravir (CAB-LA) after it was available on the market in the United States in 2021. Here, we explored the willingness to pay for CAB-LA and associated factors and price elasticity of demand (PED) of CAB-LA among men who have sex with men (MSM) in Guangxi, China.

**Methods:**

A cross-sectional study was conducted. Univariate and multivariate ordinal logistic regression analyses were performed to explore the associated factors of willingness to pay for CAB-LA. PED was used to measure the change in the number of participants willing to pay to a change in price.

**Results:**

A total of 1,006 MSM were recruited, among which 84.1% were aged between 18 and 39 years old. The median (interquartile) of the maximum amount of willing to pay for CAB-LA was 200 (100–500) Chinese yuan (CNY) per month. Most (84.2%) were willing to pay less than 600 CNY per month. The number of participants willing to pay for CAB-LA significantly increased with decrease in the price. When the price (CNY per month) decreased from 600 to 500, 500 to 400, 400 to 300, and 300 to 200, PED was 3.13, 1.64, 1.33, and 1.17, respectively. The maximum amount of willing to pay for CAB-LA was positively associated with younger age (vs. ≥40 years group, 18–24 years group, aOR: 1.94, 95% CI: 1.32–2.85; 25–39 year group, aOR: 1.70, 95% CI: 1.20–2.42), being high educated (vs. middle school or lower group, high school or college group, aOR: 1.61, 95% CI: 1.06–2.48; bachelor’s degree or above group, aOR: 2.21, 95% CI: 1.41–3.49), monthly income ≥6000 CNY (vs. <3000 CNY, aOR: 1.46, 95% CI: 1.02–2.09), being bisexual/unsure sexual orientation (vs. gay, aOR: 1.73, 95% CI: 1.24–2.43), and heard of PrEP and used (vs. never heard of PrEP, aOR: 1.76, 95% CI: 1.11–2.77).

**Conclusion:**

The maximum amount of willing to pay for CAB-LA was low in Guangxi, China. PED of CAB-LA is relatively elastic. The waived patent protection should be considered for the wide promotion of CAB-LA, and the health education should be strengthened to improve the recognition of CAB-LA.

## Introduction

Pre-exposure prophylaxis (PrEP) is a crucial preventive measure recommended by the World Health Organization in 2015 for individuals at substantial risk of HIV-1 infection (World Health Organization, 2015). As of 2019, approximately 44 countries/regions have approved the use of oral PrEP for HIV-1 prevention (Sun et al., 2022). However, in most countries/regions, it is either not covered or only partially covered by the health insurance systems (Koppe et al., 2021; Kay and Pinto, 2020; Medland et al., 2023; Shandong Government Procurement Center, 2021). Currently, tenofovir disoproxil fumarate/emtricitabine (TDF/FTC) and tenofovir alafenamide/emtricitabine (TAF/FTC) (Fields and Tung, 2021) are the two main drugs for oral PrEP. In 2022, the original prices for TDF/FTC and TAF/FTC from Gilead Sciences, Inc. in the United States were approximately $1 800 and $1 900 per month, respectively (Killelea, et al., 2022). However, the annual price of generic TDF/FTC in the United States is less than $360 (Amick et al., 2024), approximately $221 per year in the United Kingdom (Department of Health and Social Care, 2022), and ranges from $797 to $3 600 per year in most European countries (AIDS Action Europe, 2022). The additional regular check-ups, such as liver function and kidney function tests may further increase the cost. It was reported that even in those with health insurance, the out-of-pocket costs for PrEP and related laboratory tests were more than $2 000 per person per year (pppy) in the United States (Kay and Pinto, 2020). In China, TDF/FTC was not covered by health insurance with the price of 700 Chinese yuan (CNY) per month, and related laboratory tests were approximately 1,400 CNY pppy. The total cost for PrEP was approximately 9,800 CNY (∼$1,360) pppy. The prohibitive high cost and daily medication were two key barriers for oral PrEP implementation (Peng et al., 2018; Cao et al., 2020; van Dijk et al., 2021; Qu et al., 2018). Previous studies showed that even among men who have sex with men (MSM), who are at the highest risk of HIV infection, only 3.3%–39% of eligible MSM have ever used PrEP (Finlayson et al., 2019; Parmley et al., 2022; Kota et al., 2021; Sun et al., 2023). Most MSM were unwilling or only willing to pay a small fee for PrEP (Cao et al., 2020; Ko et al., 2016; Morgan et al., 2018). A study in Chengdu, China, found that 63.4% of MSM were willing to pay less than 7 344 CNY pppy for PrEP (Cao et al., 2020).

In December 2021, the United States Food and Drug Administration approved the first long-acting PrEP product called long-acting injectable cabotegravir (CAB-LA) for eligible populations at a substantial risk of HIV-1 infection (US Food and Drug Administration, 2021). The treatment regimen is given first as two initial injections administered 1 month apart and then every 2 months for as long as the risk behaviors continue. The efficacy of CAB-LA yielded a 66%–88% reduction in the rate of sex-related HIV transmission compared to TDF/FTC (HIV Prevention Trials Network, 2021; Delany-Moretlwe et al., 2022). Most previous studies focusing on the acceptability and preferences of long-acting PrEP products were conducted on its development stage and assuming free use (John et al., 2023; Schoenberg et al., 2023; Paudel et al., 2023; Meyers et al., 2018; Huang et al., 2019; Chan et al., 2022). However, the cost of CAB-LA in the United States was $22 200 pppy (Liu, 2021; Pepperrell T et al., 2022), and it was $9 275 pppy in the United Kingdom (Pepperrell T et al., 2022; NICE, 2022). The unaffordable cost will be a great challenge in most of countries/regions. A Phase II clinical trial of CAB-LA in the United States showed that participants were only willing to pay $1 272 (range from $120 to $3 600) pppy for long-acting PrEP products (Kerrigan et al., 2018). However, there was still no study focusing on willingness to pay for CAB-LA after its availability on the market. The objectives of this study are to assess the maximum amount of willing to pay for CAB-LA and associated factors and explore the price elasticity of demand (PED) of CAB-LA among MSM in Guangxi, China. The findings of this study will provide a scientific basis for the implementation of CAB-LA service planning in China.

## Methods

### Study design and study area

A cross-sectional study using face-to-face interviews was conducted in Guangxi, China. Guangxi is one of the provinces with the highest prevalence of HIV in China, with 10,000 new HIV infections reported annually from 2010 to 2020 (Lan, et al., 2021; Meng et al., 2018). The study subjects were recruited from the largest gay-friendly health consulting service center in Nanning, the capital city of Guangxi. It is a non-governmental organization established in September 2006 jointly funded by Nanning Center for Disease Prevention and Control and United States Family Health International. There are five staff, and the main missions are providing free peer counseling, testing, and prevention of HIV and sexually transmitted diseases.

### Recruitment of participants

Participants were recruited consecutively from 1 March to 30 July 2022. During the study period, clients who visited the center were invited to participate. They were also encouraged to introduce their gay friends to come and join in this study. MSM in the contact list were also invited by phone. Additionally, recruitment information was also published via two social networking apps (WeChat and BLUED) and some settings where gay activities occur (gay bars/clubs/bathhouses). The study inclusion criteria were men aged ≥18 years, self-reported as having had oral or anal sex with another man in the past year, a negative HIV test result, and a resident of Guangxi. Individuals with mental disabilities were excluded. Four center MSM peer staff were hired and trained to facilitate the recruitment and interviews.

### Sample size

Based on the sample size formula for a cross-sectional study: *n= Z*_*a*_^*2*^ *× p (1 − p)/d* ^*2*^ *×deff*, the significance level *α* was set as 0.05 and the precision *d* was set as 0.05. A study in Chengdu, China, showed that 88.9% of MSM were willing to pay for PrEP (Peng et al., 2019) and that the rate of willingness to pay *(p*) was set as 0.5 to obtain the maximum sample size. The design effect (*deff*) was set to 2 to account for the non-random sampling method. With these parameters, the sample size was 768. Assuming that approximately 10% of the participants would have incomplete data or no HIV test results, the total sample size increased to 845.

## Measurements

A structured questionnaire was used to collect information including demographic characteristics (age, ethnicity, occupation, education level, marital status, area of residence, and monthly income), sexual behaviors, sexualized drug use in the past year, HIV knowledge, HIV attitude and behavior, experience of using PrEP, willingness to use CAB-LA, and the maximum of willing to pay for CAB-LA. Knowledge of HIV was assessed by presenting participants with six questions/statements to answer: (1) AIDS is a serious infectious disease with high mortality. (2) AIDS needs lifelong treatment. (3) Can HIV be transmitted through sex? (4) MSM is the high-risk population of HIV infection. (5) List the ways that people can protect themselves from sexual transmission of HIV. (6) Correct condom use during sex can reduce the risk of HIV infection. The possible responses for item 5 were as follows: “Condom use,” “I don’t know,” or “Other.” Responses to the other five questions/statements were either “Yes” or “No.” All correct answers were considered “good knowledge of HIV” and otherwise considered “poor knowledge of HIV.” Willingness to use CAB-LA was assessed as follows: a brief introduction of CAB-LA was given to each participant, including the treatment regimen, its effectiveness, and side effects based on the results of clinical trials. Participants would also be given the opportunity to ask any questions with their concerns. After the introduction, the interviewer would ask the following question: “If CAB-LA is approved in China, and is available on the market with free of cost, would you use it?” Answers were measured on a 5-point Likert scale: 1 = definitely would, 2 = probably would, 3 = uncertain, 4 = probably would not, and 5 = definitely would not. Excluding those who answered “definitely would not,” participants were further asked the maximum amount that they were willing to pay per month (CNY) for CAB-LA if it was not free, which used an open question where the participants could fill in the price they prefer. All the participants were given a rapid HIV and syphilis screening, and those who tested positive were referred to the Nanning Center for Disease Control and Prevention for confirmation and counseling (if required).

### Data analysis

Data management was performed using EpiData version 3.1 (EpiData Association, Odense, Denmark) with double-entry and verification. Data were analyzed using R version 4.1.2. (R Foundation for Statistical Computing, Vienna, Austria). The maximum amount of willing to pay per month was classified into <100, 100–199, 200–299, 300–399, 400–499, 500–599, and ≥600 CNY seven groups based on the data distribution. Unprotected sex behavior was defined as no or inconsistent condom use in the past month. Univariate and multivariate ordinal logistic regression analyses were performed to explore the associated factors of the maximum amount of willing to pay for CAB-LA presenting crude odds ratio (OR), adjusted OR, and 95% confidence interval (95% CI). Variables with a *p*-value less than 0.1 in the univariate analysis were included in the initial multivariate analysis. The statistical significance level was set as 0.05. The Cochran–Armitage trend test and PED were used to measure a change in the proportion/number of participants willing to pay to a change in price (the maximum amount of willing to pay per month). PED was measured successively at price decreased from 600 to 500, 400, 300, and 200 CNY per month using the following formula:

$$PED=\frac{\%\;\text{Change}\;\text{in}\;\text{quantity}\;\text{demanded}\;}{\%\;\text{Change}\;\text{in}\;\text{price}}=\frac{\left(\left(Q_{N}-Q_{I}\right)/\left(Q_{N}+Q_{I}\right)/2\right),}{\left(\left(P_{N}-P_{I}\right)/\left(P_{N}+P_{I}\right)/2\right)}$$

where

Q_N_ is the new quantity demanded after the maximum amount of willing to pay decreased 100 CNY per month.
Q_I_ is the initial quantity demanded before the maximum amount of willing to pay decreased 100 CNY per month.
P_N_ is the new maximum amount of willing to pay per month.
P_I_ is the initial maximum amount of willing to pay per month.

If PED>1: which means the change in price leads to an even bigger change in demand, the product is very responsive to a change in price. If PED<1, which means the product is relatively unresponsive to a change in price. When a unitary elastic good has a change in demand which is equal to the change in price, PED = 1.

## Results

### Participant characteristics

During the study period, there were 1 152 visits to the gay-friendly health consulting service center, of which 132 were repeated visits and 14 visitors rejected to join the study. In total, 1006 eligible participants were recruited. Most participants were aged between 18 and 39 years (84.1%), of Han nationality (58.2%), single (80.5%), with a high school or higher education (90.6%), had a monthly income between 3000 and 5999 CNY (48.6%), and 19.2% of participants were students (Table 1).

**TABLE 1:** General characteristics of men who have sex with men (MSM) in Guangxi, China, 2022 (N = 1006).

Variable	N	%
Total	1006	100.0
Age group (years)		
18–24	342	34.0
25–39	504	50.1
≥40	160	15.9
Ethnicity		
Han	585	58.2
Other	421	41.8
Education level		
Middle school or lower	95	9.4
High school or 3-year college	509	50.6
Bachelor’s degree or above	402	40.0
Marital status		
Married^a^	167	16.6
Single	810	80.5
Divorced/widowed	29	2.9
Occupation		
Student	193	19.2
Factory/company employee	487	48.4
Farmer/self-employed	147	14.6
Part-time or unemployed	90	8.9
Government employee	89	8.8
Area of residence		
Rural area	428	42.5
County or township	217	21.6
Urban area	361	35.9
Monthly income (CNY)		
<3000	249	24.8
3000–5999	489	48.6
≥6000	268	26.6

^a^ Married to a woman. CNY, Chinese yuan; 1 CNY , 0.14 US, dollar ($).

Among the study subjects, 84.5% self-reported as being gay, 63.3% had non-regular sexual partners, and 30.6% reported having unprotected anal intercourse in the past month. Most participants (97.5%) ever sought male partners on the internet, and 50.9% had 2–5 male partners in the past 6 months. More than one-third (38.2%) of participants reported sexualized drug use and 6.5% were diagnosed with a sexually transmitted disease in the past year.

Of the six questions/statements about HIV knowledge, 62.5% of participants answered them all correctly. Among all participants, 65.0% perceived themselves to be at a moderate or high risk of HIV infection, 73.4% had ever heard of PrEP, but only 8.3% ever used.

### The maximum amount of willing to pay for CAB-LA and the PED

**FIGURE 1:**
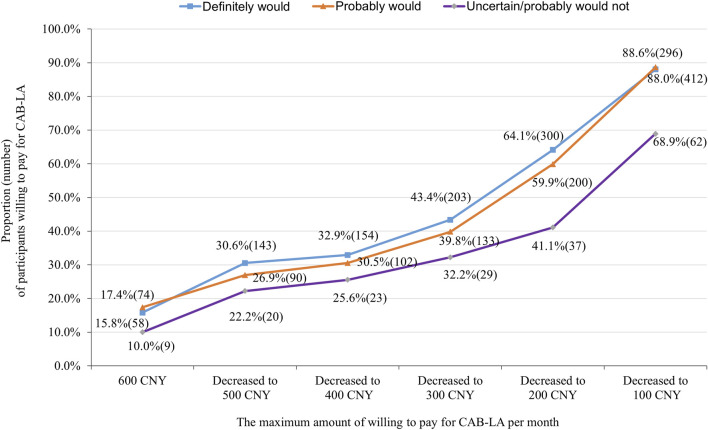
Proportion (number) of participants willing to pay for long-acting injectable cabotegravir (CAB-LA) in different prices stratified by willingness to use (separated into “definitely would,” “probably would,” and “uncertain/probably would not” use groups).

**FIGURE 2:**
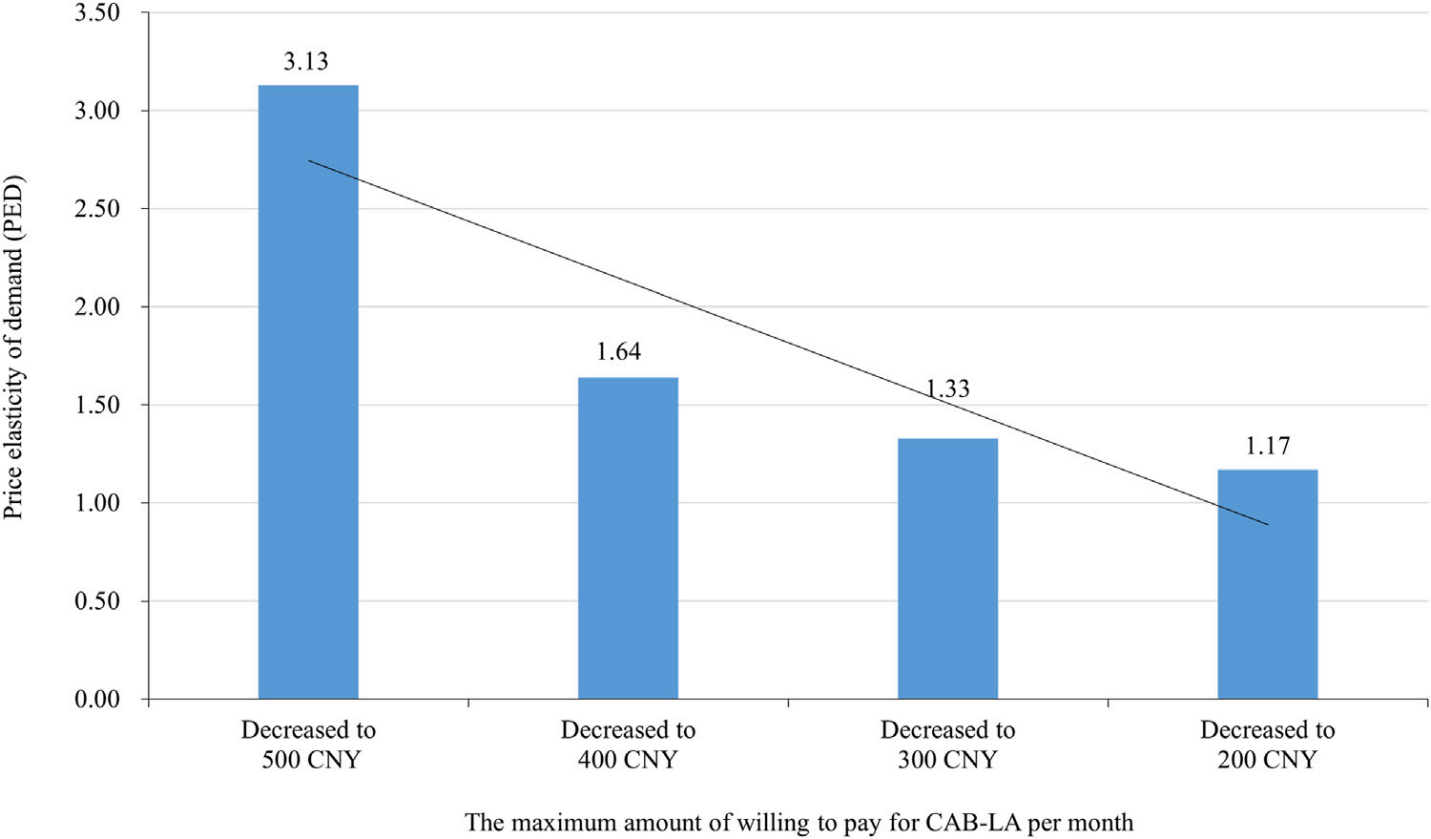
Price elasticity of demand (PED) of long-acting injectable cabotegravir (CAB-LA) in different prices (the maximum amount of willing to pay for CAB-LA per month).

Most participants (79.8% of) would use CAB-LA (definitely would: 46.5%, probably would: 33.3%, uncertain: 3.0%, probably would not: 6.1%, definitely would not: 11.1%) if it was approved and freely for use in China, excluding 112 participants who answered ‘‘definitely wouldn't,’’ 894 participants were further asked about the maximum amount of willing to pay for CAB-LA if it was not free. Two participants rejected to answer, resulting in 892 participants giving the maximum amount of willing to pay for CAB-LA. The median (interquartile) of the maximum amount of willing to pay for CAB-LA was 200 (100–500 CNY) CNY per month, with 84.2% liking to pay less than 600 CNY per month. When the cut-off points of the maximum amount of willing to pay for CAB-LA were set as 600, 500, 400, 300, 200, and 100 CNY per month, the number (proportion) of participants that was willing to pay was 141 (15.8%), 253 (28.4%), 279 (31.3%), 365 (40.9%), 537 (60.2%), and 770 (86.3%), respectively (Figure 1). The proportion of participants willing to pay was increased with decrease in the price decreased (all *p*-values <0.001), especially in the “definitely would”, and “probably would” groups (Figure 1). When the price (CNY per month) of CAB-LA decreased from 600 to 500, 500 to 400, 400 to 300, and 300 to 200, PED was 3.13, 1.64, 1.33, and 1.17, respectively (Figure 2).

### Factors associated with the maximum amount of willing to pay for CAB-LA

After classifying the maximum amount of willing to pay into <100, 100–199, 200–299, 300–399, 400–499, 500–599, and ≥600 CNY per month, age, education level, marital status, area of residence, monthly income, self-reported sexual orientation, HIV knowledge, and experience of using PrEP were significantly associated with the maximum amount of willing to pay for CAB-LA in the univariate ordinal logistic regression (Supplementary Table S1). In the multivariate ordinal logistic regression, younger age (vs. ≥40 years group, 18–24 years group: aOR: 1.94, 95% CI: 1.32–2.85; 25–39 years group: aOR: 1.70, 95% CI: 1.20–2.42), being highly educated (vs. middle school or lower group, high school or college group, aOR: 1.61, 95% CI: 1.06–2.48; bachelor’s degree or above group: aOR: 2.21, 95% CI: 1.41–3.49), monthly income ≥6000 CNY (vs. <3000 CNY, aOR: 1.46, 95% CI: 1.02–2.09), being bisexual/unsure sexual orientation (vs. gay, aOR: 1.73, 95% CI: 1.24–2.43), and heard of PrEP and used (vs. never heard of PrEP, aOR: 1.76, 95% CI: 1.11–2.77) were significantly and positively associated with the maximum amount of willing to pay for CAB-LA, while marital status, area of residence, and HIV knowledge were non-significant (Table 2).

**TABLE 2:** Factors associated with the maximum amount of willing to pay per month for CAB-LA among men who have sex with men (MSM) in Guangxi, China, 2022 (N = 892^*^).

Variable	Crude OR (95% CI)	*p*-value	Adjusted OR (95% CI)	*p*-value
Age group (years)				
18–24	2.13 (1.49–3.05)	<0.001	1.94 (1.32–2.85)	0.003
25–39	2.10 (1.50–2.97)	<0.001	1.70 (1.20–2.42)	0.001
≥40	1.00		1.00	
Education level				
Middle school or lower	1.00		1.00	
High school or college	1.97 (1.30–2.99)	0.001	1.61 (1.06–2.48)	0.027
Bachelor’s degree or above	3.19 (2.08–4.90)	<0.001	2.21 (1.41–3.49)	0.001
Monthly income (CNY)				
<3000	1.00		1.00	
3000–5999	0.75 (0.56–0.99)	0.049	0.89 (0.65–1.23)	0.479
≥6000	1.38 (0.99–1.91)	0.053	1.46 (1.02–2.09)	0.039
Self-reported sexual orientation				
Gay	1.00		1.00	
Bisexual/unsure	1.48 (1.06–2.07)	0.023	1.73 (1.24–2.43)	0.001
Experience of using PrEP				
Never heard of PrEP	1.00		1.00	
Heard of PrEP but never used	1.46 (1.11–1.92)	0.007	1.32 (0.99–1.75)	0.056
Heard of PrEP and used	2.08 (1.34–3.25)	0.001	1.76 (1.11–2.77)	0.015

^*^Only 892 participants included in the analysis as 112 participants who answered ‘‘definitely would not’’ use CAB-LA, and two participants refused to answer the maximum amount of willing to pay were excluded. The outcome variable was divided into <100, 100–199, 200–299, 300–399, 400–499, 500–599, and ≥600 CNY, per month seven levels and used multivariate ordinal logistic regression.

CAB-LA, long-acting injectable cabotegravir; OR, odds ratio. CI, confidence interval. CNY, Chinese yuan, 1 CNY , 0.14 US, dollar ($); PrEP: pre-exposure prophylaxis.

## Discussion

To our knowledge, this is the first study to assess the maximum amount of willing to pay and PED for CAB-LA among MSM in China after CAB-LA has been approved for use in the United States. Our study showed that the median of the maximum amount of willing to pay was 200 CNY per month (interquartile: 100–500 CNY) and majority of participants (84.2%) willing to pay less than 600 CNY per month for CAB-LA. Younger age, higher education level, having a high monthly income, being bisexual/unsure sexual orientation, and experience of using PrEP were positively associated with the maximum amount of willing to pay for CAB-LA. When the price was decreased from 600 to 500, 400, 300, 200, and 100 CNY per month, PED was decreased gradually.

Our study showed that 86.3% of participants were willing to pay for CAB-LA if its price was 100 CNY per month (∼$169 pppy). The median of the maximum amount of willing to pay for CAB-LA was 200 CNY per month (∼$338 pppy, interquartile: $169–$833 pppy). This finding was similar to that of a study in Chengdu, China, in 2018, which found that 82% of MSM were willing to pay over 100 CNY per month (∼$169 pppy) for PrEP (Peng et al., 2019). However, the price was lower than that in a previous study in the United States in 2018 (mean: $1272 pppy; range: $120–3 600 pppy) (Kerrigan et al., 2018), and much lower than the current market price in the United States ($22 200 pppy) (Liu, 2021), United Kingdom ($9 275 pppy) (Pepperrell T, et al., 2022; National Institute for Health and Care Excellence, 2022), and Spain ($2 474 pppy) (Aparicio et al., 2024). These results indicated that the high cost will post a great challenge to the widespread promotion of CAB-LA in China. For the affordability of CAB-LA, the waived patent protection should be considered, especially in the developing and underdeveloped countries (Moyo et al., 2022; Jenkins et al., 2023). In addition, our study showed that the demand for CAB-LA had a high response to price change, especially when the price decreased from 600 to 500 CNY per month (PED = 3.13). This indicated that price reduction will be an effective policy to promote CAB-LA in China.

In this study, we found that younger age, higher education level, higher monthly income, and heard of PrEP and used were positively associated with the maximum amount of willing to pay for CAB-LA. The study subjects who were of younger age (Mpunga et al., 2021), with higher education level (Restar et al., 2022), and heard of PrEP and used (Guo et al., 2023) might have a better comprehension for long-acting PrEP and more concerned about their self-protection (Kota et al., 2021; Rao et al., 2019; Mansergh et al., 2021), and participants with higher education level and higher monthly income might be able to pay higher payments (Chan et al., 2022; Morgan et al., 2018). In addition, those self-reported bisexual/unsure sexual orientation MSM were willing to pay higher price for CAB-LA than gay men. This may because they are more likely to have sexual intercourse with their wives and have a responsibility to protect their families. It may also be because they had a higher risk of HIV infection (Yi et al., 2019) and were more willing to use PrEP (Karuga et al., 2016).

This study had some limitations. First, we recruit participants from the largest gay health consulting service center in Guangxi. MSM who did not routinely visit this center would not be recruited, resulting in potential sampling bias. Second, reporting bias may have occurred as sensitive questions were asked via face-to-face interview. Finally, we measured some key variables with a single item, such as the perceived risk of HIV infection.

## Conclusion

The maximum amount of willing to pay for CAB-LA was low in Guangxi, China. Associated factors included age, education level, monthly income, self-reported sexual orientation, and experience of using PrEP. PED of CAB-LA is relatively elastic. The waived patent protection should be considered for the wide promotion of CAB-LA, and the health education should be strengthened to improve the recognition of CAB-LA.

## Data Availability

The original contributions presented in the study are included in the article/Supplementary Material; further inquiries can be directed to the corresponding author.
